# The role of tocilizumab in the treatment of post‐transfusion hyperhaemolysis

**DOI:** 10.1002/jha2.779

**Published:** 2023-10-23

**Authors:** Naeem Desai, Jayne Peters, Elizabeth Davies, Joseph Sharif

**Affiliations:** ^1^ Haematology Manchester Royal Infirmary Manchester UK; ^2^ Department of Haematology NHS Blood and Transplant Manchester UK

**Keywords:** cytokines, haemolysis, sickle cell anaemia, sickle cell disease

## Abstract

Hyperhaemolysis syndrome (HHS) is a serious complication of transfusion mostly reported in patients with sickle cell disease. HHS is characterised by the destruction of both donor and autologous red blood cells. Tocilizumab is a recombinant humanised monoclonal antibody that inhibits the binding of interleukin‐6 and has been used in the treatment of severe/critical coronavirus disease 2019 infection but also some cases of HHS. We describe two further cases of HHS successfully treated with tocilizumab and propose a decision aid for when to consider this treatment.

## 1 INTRODUCTION

The pathogenesis of hyperhaemolysis syndrome (HHS) remains unclear; proposed mechanisms include complement‐mediated ‘bystander’ haemolysis in the context of alloimmunisation and macrophage activation resulting in peripheral consumption of erythrocytes [1, 2].

In England, eculizumab is recommended for both delayed haemolytic transfusion reaction (DHTR) and HHS where first‐line treatment has failed with ongoing haemolysis [3, 4]. Based on the hypothesis of macrophage activation, tocilizumab, an interleukin (IL)‐6 receptor blocker, has recently been used in a small number of cases of HHS [5, 6]. HHS can present with similar clinical features to a DHTR [7].

## 2 CASE 1

**FIGURE 1:**
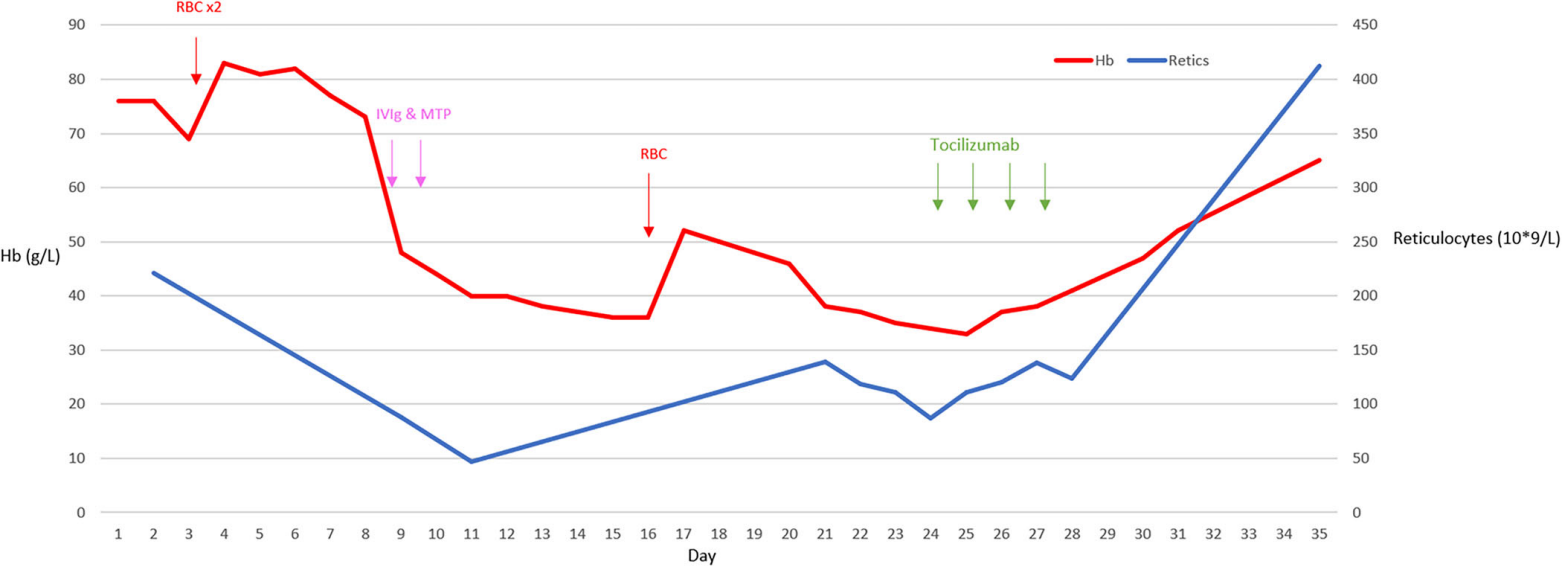
X‐axis: days from admission; composite y‐axis representing haemoglobin (Hb), reticulocyte count (retics). Vertical arrows representing timing of blood transfusion (RBC), intravenous immunoglobulin (IVIg) and tocilizumab.

**FIGURE 2:**
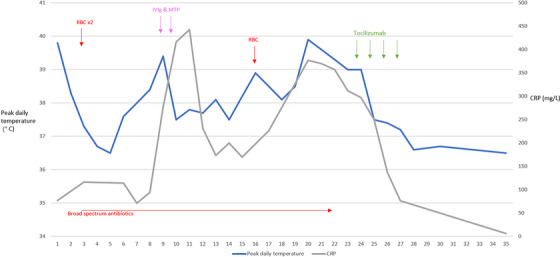
X‐axis: days from admission; composite y‐axis representing peak daily temperature and CRP (C‐reactive protein). Vertical arrows representing timing of blood transfusion (RBC), intravenous immunoglobulin (IVIg) and tocilizumab.

A 21‐year‐old male with HbSS received a 2‐unit red blood cell (RBC) transfusion during an admission with a vaso‐occlusive crisis. Five days later he became acutely unwell with generalised bone pain, tachycardia, fever, haemoglobinuria and a haemoglobin of 47 g/L. He was treated with intravenous immunoglobulin (IVIg) 1 g/kg for 2 days and methylprednisolone 500 mg IV for 2 days. Haemoglobin continued to decline to a nadir 36 g/L over the subsequent 5 days and he was transfused a further 1‐unit RBC. Within a few days, the haemoglobin level declined from 52 to 33 g/L with a relative reticulocytopenia (see Figure 1). HPLC results demonstrated persistence of HbA (32%). Red cell immunohematology investigations did not identify a new alloantibody by indirect antiglobulin test (IAT) or enzyme and the direct antiglobulin test (DAT) was 1+ (IgG). The patient remained unwell with generalised pains, high fevers and persistent tachycardia. There were no signs of localised infection and therefore empirical antibiotics were discontinued. A decision was made to give tocilizumab at 8 mg/kg for 4 days in lieu of eculizumab. Within 12 h of the first dose there was an improvement in the clinical and laboratory parameters including resolution of pain, fever, and tachycardia (see Figure 2). Over the subsequent days there was a steady rise in reticulocytes and haemoglobin, and he was discharged the following week, subsequent bloods tests demonstrating complete recovery to baseline haemoglobin. Ferritin improved to 4924 μg/L from a peak of 52,211 μg/L prior to IVIg and corticosteroid; the patient had a high baseline ferritin due to a history of multiple transfusions.

## 3 CASE 2

A female in her 30s with HbSS underwent urgent red cell exchange due to a suspected stroke. Prior to transfusion, she received rituximab 375 mg/m^2^ and prednisolone 1 mg/kg due to a history of recurrent HHS. During transfusion she became acutely unwell with all‐over body pain, tachycardia and haemoglobinuria; transfusion was immediately stopped after approximately 2 units and the patient was treated for suspected HHS with eculizumab 900 mg. Six days post‐transfusion the patient became acutely unwell with fever, generalised bone pain, haemoglobinuria with a drop in haemoglobin to 39 g/L. HPLC demonstrated the persistence of HbA at 31%. Red cell immunohaematology investigations did not identify a new alloantibody by IAT or enzyme and the DAT was 1+ (IgG). The patient was treated for HHS with IVIg 1 g/kg and methylprednisolone 500 mg IV. The following day, the haemoglobin concentration was 29 g/L. Eculizumab 900 mg was given for ongoing severe haemolysis and the patient was transfused 1 unit RBC. Immediately following the transfusion, the patient developed frank haemoglobinuria, fever, and generalised bone pain.

**FIGURE 3:**
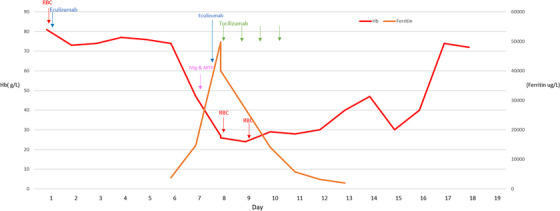
X‐axis: days from admission; composite y‐axis representing haemoglobin (Hb) and serum ferritin levels. Vertical arrows representing timing of blood transfusion (RBC), intravenous immunoglobulin (IVIg) and MTP (methylprednisolone), eculizumab and tocilizumab.

Tocilizumab was administered at a dose of 8 mg/kg and resulted in an immediate clinical response including improvement in vital signs and resolution of frank haemoglobinuria. There was a marked reduction in serum ferritin from 47,000 μg/L (pre‐dose) to 40,000 μg/L immediately post‐dose and to 27,000 μg/L the following day. The repeat haemoglobin the following day remained critically low at 24 g/L (see Figure 3). Due to severe drowsiness and concern of cerebral hypoperfusion a further one‐unit RBC was administered. The patient again experienced severe generalised bone pain, fever, and frank haemoglobinuria. The transfusion was immediately discontinued, and a second dose of tocilizumab was administered associated with a rapid clinical improvement in vital signs and pain, resolution of haemoglobinuria within hours and reduction in serum ferritin to 14,000 μg/L. She received 2 further doses of tocilizumab over the next 2 days with continuous improvement in haemolytic markers, reticulocyte count and serum ferritin. The patient continued to improve clinically over 7 days with an increase in haemoglobin to a peak of 97 g/L. There was also a marked improvement in neurological symptoms with no gross deficit and the patient was discharged home.

## 4 DISCUSSION

The use of tocilizumab in these two cases was associated with rapid improvement in clinical and biochemical features of inflammation and recovery in haemoglobin and reticulocytes. Tocilizumab is widely used for the treatment of chimeric antigen receptor (CAR) T‐cell‐induced cytokine release syndrome and in other rheumatological and inflammatory conditions [8, 9, 10]. It has also been associated with improved outcomes in coronavirus disease 2019 infection and appears to be well tolerated [11, 12, 13]. IL‐6 is the main proinflammatory cytokine resulting in C‐reactive protein (CRP) generation and tocilizumab has been associated with lower CRP levels and therefore caution is recommended interpreting CRP values within the clinical context [14].

**FIGURE 4:**
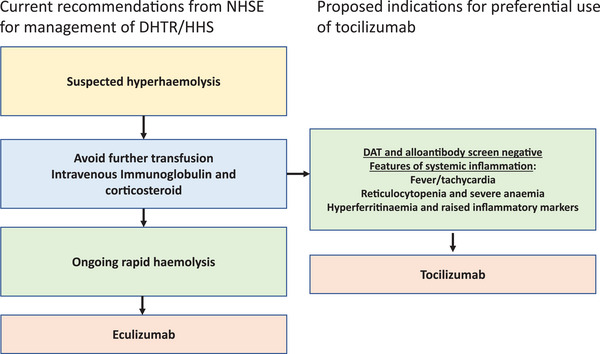
Management algorithm for the management of delayed haemolytic transfusion reaction/hyperhaemolysis syndrome (DHTR/HHS) as per NHS England with proposed amendments to include tocilizumab.

We propose that in cases of HHS with predominant features of systemic inflammation and without laboratory features of DHTR, tocilizumab should be considered (see Figure 4). HHS in SCD is associated with significant morbidity and has implications for future transfusion. Strategies to prevent alloimmunisation and haemolytic transfusion reactions are essential [15].

Further understanding of the underlying pathophysiology of HHS is required to aid diagnosis and classification as well as guide treatment. National registries and haemovigilance schemes must capture all pertinent details of cases to facilitate further research in this at‐risk patient group.

## AUTHOR CONTRIBUTIONS

Naeem Desai and Joseph Sharif collected the data. Naeem Desai, Jayne Peters, Elizabeth Davies and Joseph Sharif wrote the manuscript.

## CONFLICT OF INTEREST STATEMENT

The authors declare no conflict of interest.

## FUNDING INFORMATION

The authors received no specific funding for this work.

## ETHICS STATEMENT

Ethical approval is not required for this study.

## PATIENT CONSENT STATEMENT

Informed, written consent for the publication was obtained from the patients.

## CLINICAL TRIAL REGISTRATION

N/A.

## Data Availability

For original data, please contact naeemdesai@nhs.net.

## References

[1] Win N . Hyperhemolysis syndrome in sickle cell disease. Expert Rev Hematol. 2009;2(2):111–5. 21083443 10.1586/ehm.09.2

[2] Danaee A , Inusa B , Howard J , Robinson S . Hyperhemolysis in patients with hemoglobinopathies: a single‐center experience and review of the literature. Transfus Med Rev. 2015;29(4):220–30. 26209603 10.1016/j.tmrv.2015.06.001

[3] Chou ST , Alsawas M , Fasano RM , Field JJ , Hendrickson JE , Howard J , et al. American Society of Hematology 2020 guidelines for sickle cell disease: transfusion support. Blood Adv. 2020;4(2):327–55. 31985807 10.1182/bloodadvances.2019001143 PMC6988392

[4] NHS England Clinical Commissioning Policy . Rituximab and eculizumab for the prevention and management of delayed haemolytic transfusion reactions and hyperhaemolysis in patients with haemoglobinpathies. https://www.england.nhs.uk/publication/rituximab‐and‐eculizumab‐for‐the‐prevention‐and‐management‐of‐delayed‐haemolytic‐transfusion‐reactions‐and‐hyperhaemolysis‐in‐patients‐with‐haemoglobinopathies/ Accessed 21 June 2023

[5] Menakuru SR , Priscu A , Dhillon V , Salih A . Acute hyperhemolysis syndrome in a patient with known sickle cell anemia refractory to steroids and IVIG treated with tocilizumab and erythropoietin: a case report and review of literature. Hematol Rep. 2022;14(3):235–9. 35893156 10.3390/hematolrep14030032 PMC9326715

[6] Meenan J , Hall R , Badle S , Chatterjee B , Win N , Tsitsikas DA . Tocilizumab in the management of posttransfusion hyperhemolysis syndrome in sickle cell disease: The experience so far. Transfusion 2022;62(3):546–50. 35092617 10.1111/trf.16805

[7] Chen F , Booth C , Barroso F , Bennett S , Kaya B , Win N , Telfer P . Salvage of refractory post‐transfusion hyperhaemolysis by targeting hyperinflammation and macrophage activation with tocilizumab. Transfus Med. 2022;32(5):437–40. 34046955 10.1111/tme.12793

[8] European Medicines Agency . RoActemra (Tocilizumab) Summary of product characteristics. https://www.ema.europa.eu/en/medicines/human/EPAR/roactemra. 2016. Accessed 21 June 2023.

[9] La Rosée P , Horne A , Hines M , von Bahr Greenwood T , Machowicz R , Berliner N , et al. Recommendations for the management of hemophagocytic lymphohistiocytosis in adults. Blood 2019;133(23):2465–77. 30992265 10.1182/blood.2018894618

[10] NHS England Clinical Commissioning Policy . Tocilizumab for the treatment of adult‐onset Still's disease refractory to second‐line therapy (adults) [210801P] (URN: 1609). https://www.england.nhs.uk/wp‐content/uploads/2021/08/1609‐Tocilizumab‐for‐AOSD‐Final‐August‐2021‐.pdf Accessed 21 June 2023

[11] Gupta S , Padappayil RP , Bansal A , Daouk S , Brown B . Tocilizumab in patients hospitalized with COVID‐19 pneumonia: systematic review and meta‐analysis of randomized controlled trials. J Invest Med. 2022;70(1):55–60. 10.1136/jim-2021-002001 PMC8476233 34561232

[12] Frigault MJ , Nikiforow S , Mansour MK , Hu ZH , Horowitz MM , Riches ML , et al. Tocilizumab not associated with increased infection risk after CAR T‐cell therapy: implications for COVID‐19? Blood 2020;136(1):137–9. 32457999 10.1182/blood.2020006216 PMC7332891

[13] Samec MJ , Rakholiya J , Langenfeld H , Crowson CS , Abril A , Wang B , et al. Relapse risk and safety of long‐term tocilizumab use among patients with giant cell arteritis: a single‐enterprise cohort study. J Rheumatol. Published online: Jun 15, 2023. 10.3899/jrheum.2022-1214 PMC10543396 37321636

[14] Berman M , Berliner S , Bashouti N , Elkayam O , Ziv‐Baran T . Reduced C‐reactive protein level at hospital admission in patients treated with tocilizumab–an attention may be required. SSRN 4263956. 10.1016/j.heliyon.2023.e16665 PMC10245240 37292345

[15] Gardner K , Hoppe C , Mijovic A , Thein SL . How we treat delayed haemolytic transfusion reactions in patients with sickle cell disease. Br J Haematol. 2015;170(6):745–56. 25967919 10.1111/bjh.13494

